# Decoding cardiorenal crosstalk: Intercellular communication mechanisms and therapeutic insights in cardiorenal syndrome

**DOI:** 10.1016/j.isci.2026.115848

**Published:** 2026-06-26

**Authors:** Miaomiao Wei, Yuxiao Cao, Lei Wei, Aolin Li, Haomin Zhang, Jiaxin Cui, Yuxin Kang, Zhihan Yang, Guohua Shi, Lu Fan, Junping Zhang, Xuezheng Liu, Yanyang Li, Xiaoling Wang, Shichao Lv

**Affiliations:** 1 Department of Internal Medicine, First Teaching Hospital of Tianjin University of Traditional Chinese Medicine, National Clinical Research Center for Chinese Medicine Acupuncture and Moxibustion, Tianjin 300380, China; 2 Department of Integrated Traditional Chinese and Western Medicine, Tianjin Medical University Cancer Institute & Hospital, National Clinical Research Center for Cancer, Tianjin 300060, China; 3 Cardiology, Qianan Hospital of Traditional Chinese Medicine, Tangshan, Hebei 064400, China

**Keywords:** Biological sciences

## Abstract

Cardiorenal syndrome (CRS) is a pathological condition characterized by the synergistic deterioration of cardiac and renal function. This review attempts to systematically elucidate the pathophysiological mechanisms of CRS from the perspective of intercellular communication between cardiac and renal cells. We first analyze the characteristics of communication between the heart and kidneys under physiological conditions and then describe the pathological communication pathways mediated by intercellular communication (such as soluble factors and extracellular vesicles) in cardiorenal syndrome. We then provide a detailed analysis of the unique communication features observed in different subtypes of CRS. Based on this, we reinterpret the role of existing therapies in regulating cardio-renal cell communication and propose potential targeted strategies, including extracellular vesicle therapy and microRNA (miRNA)-targeted delivery systems. Finally, we discuss the challenges facing this field and future research directions, with the aim of providing new insights into the mechanisms and treatment of CRS.

## Graphical abstract

**Figure:**
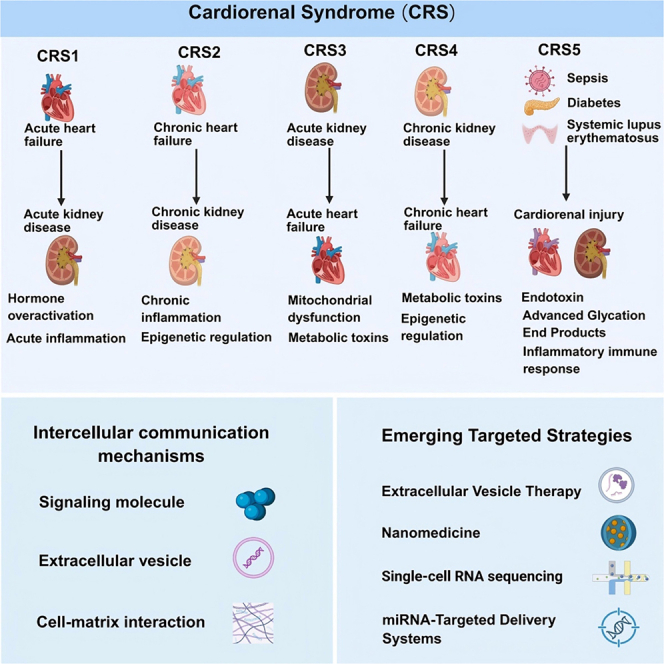

## Introduction

Cardiorenal syndrome (CRS) primarily involves the heart and kidneys, representing a syndrome where dysfunction in one organ triggers dysfunction in another.^1^ Under specific circumstances, this syndrome may lead to systemic multi-organ dysfunction and induce systemic pathological changes.^2^^,^^3^ Based on the rate of onset and the organs affected by the initial symptoms, CRS can be classified into five main types^4^: CRS type 1 emphasizes acute renal impairment triggered by acute cardiac dysfunction,^5^ such as acute kidney injury resulting from acute heart failure or cardiogenic shock. This is primarily associated with inadequate cardiac output, reduced renal perfusion, and neurohormonal activation.^6^ CRS type 2 denotes chronic renal insufficiency triggered by chronic cardiac dysfunction, commonly observed in the progression from chronic heart failure to chronic renal failure. Its core pathophysiological mechanisms involve inflammation, oxidative stress, and excessive neurohormonal activation.^7^ CRS type 3 involves acute cardiac events triggered by acute kidney injury, encompassing uremic heart failure or arrhythmias secondary to uremia. This type is closely associated with volume overload, uremic toxins, and electrolyte disturbances.^8^ Chronic heart failure resulting from chronic kidney disease falls under CRS type 4. Examples include the well-known left ventricular hypertrophy and myocardial fibrosis induced by chronic glomerulonephritis, where the pathological process often involves metabolic toxin accumulation, mitochondrial damage, and inflammatory injury.^9^ CRS type 5 represents a broad spectrum of pathologies, commonly observed in systemic diseases such as metabolic disorders, sepsis, and systemic lupus erythematosus (SLE),^10^ leading to synchronous cardiac and renal injury.^11^ A longitudinal study involving 1,000 heart failure (HF) patients revealed that approximately 70% of HF patients also have renal impairment. As chronic kidney disease (CKD) progresses, the risk of cardiovascular disease increases: 33.3%–37.1% of patients with mild to moderate CKD and 39.9% of those with moderate to severe CKD die from cardiovascular disease.^12^ CRS is characterized by high incidence, high hospitalization rates, and poor prognosis,^13^ demanding urgent clinical attention. In clinical practice, CRS patients often face multiple challenges including multi-organ decline, limited exercise tolerance, adverse effects from polypharmacy, and heavy treatment burdens.^14^ This paper, building upon established mechanisms of CRS involving hemodynamics,^15^ neuroendocrine regulation,^16^ inflammation, and oxidative stress,^17^ focuses on cellular and molecular interactions to further elucidate the pathogenesis of CRS and explore potential approaches for clinical treatment.

## Primary forms of cellular communication

**Figure 1:**
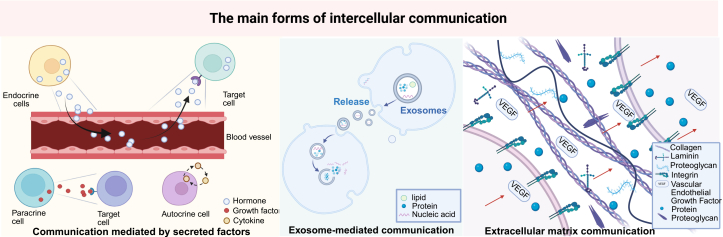
Modes of intercellular communication Left (secretory-factor-mediated cell communication): this is the most classic form of cell communication: endocrine cells secrete hormones that travel through the bloodstream to act on target cells at a distance; paracrine cells release growth factors to locally regulate neighboring cells; and autocrine cells use cytokines to regulate their own signaling. These three types of secretory signals work together to coordinate physiological activities throughout the body and at the local level. Middle (exosome-mediated cell communication): cells secrete exosomes—vesicles containing lipids, proteins, and nucleic acids—to safely deliver signaling molecules to target cells, enabling long-distance, precise information transfer. This process plays a critical role in disease regulation and tissue repair. Right (extracellular-matrix-mediated cell-cell communication): cells bind to the extracellular matrix (collagen, laminin, etc.) via surface integrins. The matrix simultaneously stores and releases growth factors such as VEGF, establishing bidirectional signaling between cells and their microenvironment. This regulates cell adhesion, proliferation, and differentiation, serving as a critical regulatory mechanism for tissue homeostasis and disease progression.

Intercellular communication is a core biological process of information transfer and functional coordination between cells,^18^ mainly including the following three basic forms: (1) chemical signal communication, which transmits signals through signaling molecules^19^ (such as hormones and cytokines) via the circulatory system or local microenvironments through endocrine, paracrine, and autocrine mechanisms to regulate the physiological activities of target cells^20^^,^^21^; this communication mediated by secreted factors is the most typical form of chemical signal communication; (2) extracellular-vesicle-mediated communication, represented by exosomes,^22^ which transports biomolecules such as proteins and nucleic acids over long distances to achieve intercellular and even transorgan information exchange^23^; and (3) extracellular matrix (ECM)-mediated communication,^24^ in which receptors such as integrins sense the mechanical and biochemical signals of the ECM^25^ and dynamically remodel the ECM via matrix metalloproteinases, thereby indirectly regulating cellular behavior and tissue homeostasis.^26^ Under physiological conditions, the above communication forms coordinate precisely to maintain the functional balance of tissues and organs; in pathological processes such as CRS, their synergistic function is impaired and signal transduction is disrupted, which in turn drives the occurrence and progression of CRS (Figure 1).

## Communication between cardiac and renal cells under physiological conditions

Under physiological conditions, cells in the heart and kidneys maintain a precise and dynamic bidirectional dialogue through multiple mechanisms, jointly regulating the stability of the body’s internal environment. This dialogue primarily relies on chemical signals and metabolic substances.

First, hormonal signaling serves as the core bridge in heart-kidney communication. When myocardial cells detect pressure changes during diastole, they release peptide hormones such as atrial natriuretic peptide and brain natriuretic peptide (BNP). These messengers travel via the circulation to the kidneys, where they act on the renal tubular epithelial cells to promote water and sodium excretion. This process reduces cardiac volume load and regulates blood pressure.^27^ The kidneys are not passive recipients of signals. They degrade BNP via the natriuretic peptide receptor C and neutral endopeptidase,^28^ while simultaneously secreting apical kidney extracts that negatively regulate cardiac BNP release.^29^ Beyond the natriuretic peptide system, nitric oxide (NO) serves as another crucial messenger. Primarily synthesized by endothelial NO synthase, NO influences the cardiovascular system by regulating vascular tone, platelet function, and neurotransmission. Research indicates that NO can reduce the intensity and frequency of myocardial cell contractions while promoting myocardial cell relaxation. These seemingly inhibitory effects actually play a crucial role in conserving energy and protecting the heart under both physiological and pathological conditions.^30^ NO also finely modulates renal tubular transport functions in the kidneys.^31^ Reduced NO bioactivity may contribute to aging, metabolic disorders, and adverse events involving the kidneys and cardiovascular system.^32^

The renin-angiotensin-aldosterone system (RAAS) also participates in cardio-renal regulation, which is initiated by renin secretion from juxtaglomerular cells and ultimately leads to the generation of angiotensin II (Ang II).^33^ Ang II binds to angiotensin II receptors (AT1 receptors) on the surface of adrenal cortex zona glomerulosa cells, stimulating aldosterone secretion.^34^ This, in turn, regulates water-electrolyte balance and circulating blood volume via mineralocorticoid receptors on distal tubular epithelial cells.^35^ Ang II also acts on cardiomyocytes and vascular smooth muscle cells by promoting sympathetic release of norepinephrine,^36^ participating in the regulation of cardiac structure and function,^37^ thereby achieving holistic control over cardiorenal function.

Finally, metabolic substances also participate in the heart-kidney dialogue. Lactate, serving as a crucial messenger and energy carrier facilitating metabolic signaling between multiple organs including the heart and kidneys, is produced in large quantities during skeletal muscle hypoxia or high-intensity work. It is then transported via the bloodstream to supply energy to cardiomyocytes. Another portion is delivered to the liver and kidneys, where it is resynthesized into glucose through gluconeogenesis.^38^^,^^39^ Renal gluconeogenesis primarily occurs within proximal tubule epithelial cells.^40^ Additionally, ketone bodies serve as a vital energy substrate for the heart, providing an alternative energy source for myocardial cells.^41^^,^^42^ They may indirectly influence myocardial cell function and structure and also affect cardiac health by regulating the functions of intestinal epithelial cells and vascular endothelial cells.^43^ The kidneys play a dual role in ketone body metabolism. When circulating ketone concentrations are low, they are reabsorbed via the sodium-coupled monocarboxylate transporter 1 and 2 on proximal tubule epithelial cells to prevent energy loss.^44^ However, when ketone concentrations exceed the renal threshold, filtration exceeds reabsorption capacity, leading to ketonuria.^44^ The coordinated processing and conversion of lactate and ketone bodies by the heart and kidneys jointly maintain the body’s energy substrate homeostasis, adapting to dynamic changes in metabolic demands.

## Intercellular communication mechanisms in CRS

### Signaling-molecule-mediated communication

**Figure 2:**
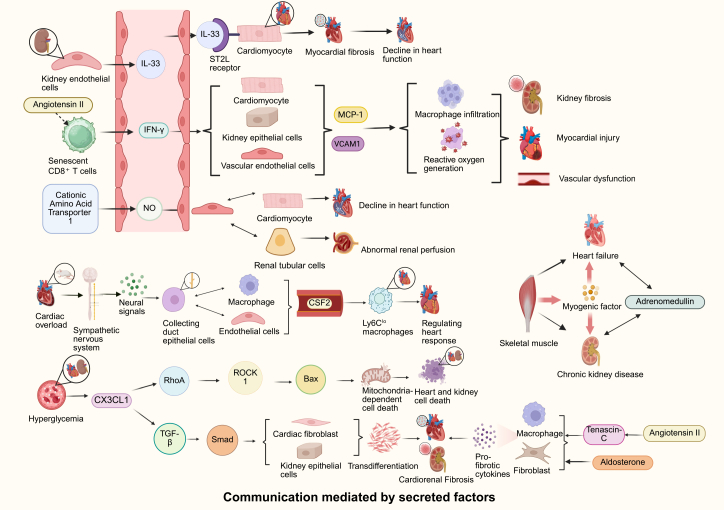
Signaling-molecule-mediated heart-kidney communication This figure summarizes the bidirectional damage communication mechanism between the heart and kidneys in cardiorenal syndrome, which is mediated by various signaling molecules including inflammatory factors, vasoactive factors, chemokines, myokines, and adrenomedullin. It systematically illustrates the pathological damage pathways of CRS mediated by these signaling molecules.

In CRS, signaling-molecule-mediated communication forms the core network for long-distance inter-organ dialogue (Figure 2). Among these, the chronic, persistent activation of inflammatory factors represents the most prominent pathological feature. Acute kidney injury stimulates rapid release of interleukin-33 (IL-33) from renal endothelial cells. Circulating IL-33 binds to transmembrane form of suppression of tumorigenicity 2 receptors on cardiomyocytes, further inducing cardiac hypertrophy, fibrosis, and impaired cardiac function.^45^ Activation of NOD-like receptor family pyrin domain-containing 3 (NLRP3) inflammasomes significantly increases production of proinflammatory cytokines, e.g., IL-1β and IL-18 in cardiac and renal cells, thereby accelerating disease progression.^46^ Aging CD8^+^ T cells enhance interferon gamma (IFN-γ) secretion upon Ang II stimulation. Circulating IFN-γ acts on renal epithelial cells, vascular endothelial cells, and cardiomyocytes, inducing expression of adhesion molecules like vascular cell adhesion molecule 1 and the inflammatory factor monocyte chemoattractant protein 1 (MCP-1). This promotes macrophage infiltration and reactive oxygen species production, exacerbating renal fibrosis, vascular dysfunction, and myocardial injury.^47^

Vasoactive factors also play a key role in the pathophysiology of heart and kidney diseases. NO is synthesized from arginine by NO synthase (NOS), with arginine uptake mediated by cationic amino acid transporter-1 (CAT-1). Impairment of this pathway reduces NO bioavailability, leading to endothelial dysfunction.^48^ This disrupts signaling between cardiomyocytes and vascular endothelial cells, resulting in abnormal myocardial perfusion and impaired cardiac function. Simultaneously, reduced NO disrupts signaling between renal vessels and tubular cells, impairing renal perfusion and sodium/water excretion.^49^ Thus, maintaining normal NO bioavailability is critical for cardiac and renal homeostasis.

Chemokines such as Fractalkine (CX3CL1) exhibit sustained overexpression in the heart and kidneys under hyperglycemic conditions. In vitro studies demonstrate that soluble CX3CL1 induces mitochondrial-dependent cell death in cardiac and renal cells by activating the RhoA/ROCK1-Bax pathway. It also promotes phenotypic transdifferentiation of cardiac fibroblasts and renal tubular epithelial cells via transforming growth factor β (TGF-β)/Smad signaling, thereby exacerbating the fibrotic progression of CRS.^50^

Myokines secreted by skeletal muscle are considered key pathological messengers linking chronic CKD and HF, forming the “kidney-skeletal muscle-heart” axis. In HF models and patients, widespread dysregulation of myokines has been observed in association with left ventricular dysremodeling. Concurrently, alterations in specific cardiac-related myokines are also present in CKD patients. Collectively, these findings indicate skeletal muscle participates in cardio-renal interactions via myokines.^51^ Furthermore, adrenomedullin (ADM), a peptide primarily produced by adrenal medulla, vascular endothelial cells, and the heart, is implicated in the pathophysiology of CRS. Associated with stress and volume overload, ADM not only serves as a significant predictor of congestive heart failure^52^ but also exhibits close links to the onset and progression of CKD.^53^

Granulocyte-macrophage colony-stimulating factor (CSF2) participates in the pathogenesis of CRS through the “heart-nerve-kidney” axis. In mouse models of cardiac pressure overload, sympathetic activation transmits neural signals to renal collecting duct (CD) epithelial cells. Activated CD cells undergo a series of intercellular interactions with tissue macrophages and endothelial cells, leading to the secretion of CSF2. Once circulating, CSF2 stimulates Ly6C^lo^ macrophages residing in the heart, thereby regulating cardiac adaptive responses.^54^

Finally, Ang II and aldosterone, as components of the RAAS, synergistically drive functional dysregulation and structural remodeling in cardiac and renal tissues.^55^ Ang II activates macrophages and fibroblasts to produce abundant pro-fibrotic cytokines by upregulating the matrix protein tenascin-C.^56^ Its downstream AT1R/p38 MAPK pathway is a key driver of cardiac and renal fibrosis.^55^^,^^57^ Aldosterone further amplifies fibrotic signaling by recruiting macrophages, activating fibroblast activation, and directly stimulating multiple pro-fibrotic factors including TGF-β, connective tissue growth factor, and endothelin-1,^55^ thereby contributing to the pathological progression of CRS.

### Extracellular-vesicle-mediated communication

**Figure 3:**
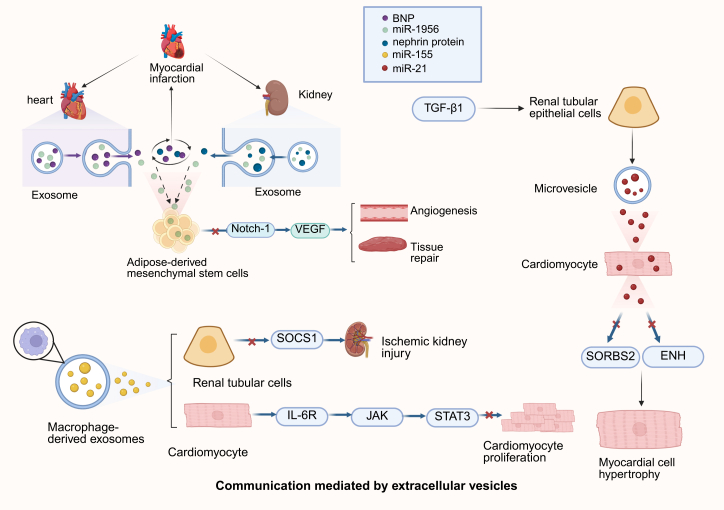
Extracellular-vesicle-mediated heart-kidney communication This figure systematically illustrates the extracellular vesicles secreted by various cell types (cardiomyocytes, renal tubular epithelial cells, and macrophages), which mediate bidirectional communication between the heart and kidneys by carrying miRNAs, proteins, and other molecules, thereby contributing to the pathological damage associated with cardiorenal syndrome.

Extracellular vesicles (EVs) serve as crucial mediators for bidirectional communication between the heart and kidneys (Figure 3). First, EVs play a pivotal role in cardiorenal crosstalk by carrying specific proteins. Gao et al. demonstrated that cardiac marker BNP and renal marker nephrin protein were significantly elevated in serum exosomes from mice after myocardial infarction,^58^ suggesting that the heart and kidneys may release specific signals into the circulation via exosomes to mediate pathological inter-organ communication. Another prospective cohort study first confirmed that cystatin C and cluster of differentiation 14 (CD14) in EVs serve as common biomarkers for cardiac and renal dysfunction.^59^ This finding suggests that EVs and their proteins participate in the multi-organ failure process of cardiorenal syndrome, while also providing new targets for developing EV-proteomics-based therapeutic strategies.

Second, EVs can also mediate cardiorenal pathogenesis through microRNAs (miRNAs). Di J et al. revealed that^58^ in CRS, TGF-β1 stimulates renal tubular epithelial cells to secrete microvesicles (MVs) containing *miR-21*. These MVs are taken up by cardiomyocytes via the bloodstream, leading to elevated levels of *miR-21* within the cardiomyocytes. Elevated *miR-21* induces cardiomyocyte hypertrophy by inhibiting the functions of the *SORBS2* and *ENH* genes. Another study demonstrated that following myocardial infarction, heart and kidney tissues release *miR-1956*-containing exosomes into the bloodstream. These exosomes can be taken up by adipose-derived mesenchymal stem cells (MSCs), which then suppress *notch homolog 1* gene activation to promote the vascular endothelial growth factor (VEGF) pathway, thereby enhancing angiogenesis and tissue repair.^60^^,^^61^ Furthermore, studies suggest that exosomes derived from M1 macrophages can serve as carriers for *miR-155*, facilitating intercellular signaling and contributing to multi-organ injury processes: on one hand, the *miR-155* they carry is transmitted between macrophages and renal tubular cells, where it targets and suppresses suppressor of cytokine signaling 1 expression, thereby exacerbating ischemic kidney injury^62^; on the other hand, *miR-155* delivered by these exosomes can target the IL-6R/JAK/STAT3 signaling pathway in cardiomyocytes, thereby inhibiting cardiomyocyte proliferation after myocardial infarction and impeding cardiac repair processes.^63^

### Extracellular-matrix-mediated communication

**Figure 4:**
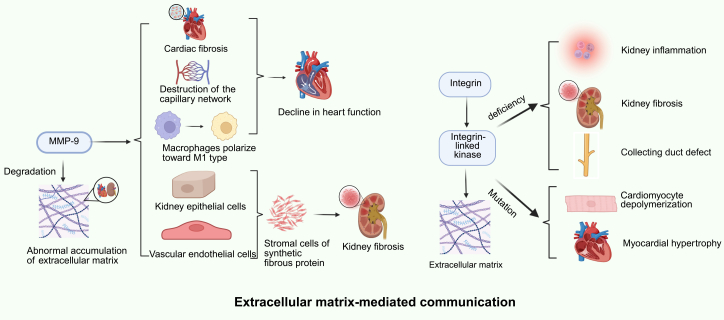
Extracellular-matrix-mediated heart-kidney communication This figure illustrates how an imbalance in extracellular matrix homeostasis (disrupted degradation mediated by MMP-9 and abnormal cell-matrix communication mediated by integrins) contributes to bidirectional damage to both the heart and kidneys through pathways such as inflammation and fibrosis, thereby playing a central role in the pathogenesis and progression of cardiorenal syndrome.

ECM remodeling is a critical component of cardiorenal communication^64^ (Figure 4). Matrix metalloproteinase-9 (MMP-9) plays a central role in this process. Under CRS conditions, abnormal ECM accumulation leads to irreversible decline in cardiac and renal function, and MMP-9 possesses ECM-degrading capabilities. Additionally, MMP-9 activates pro-fibrotic signaling pathways such as TGF-β, contributing to CRS pathogenesis. In the heart, MMP-9 exacerbates cardiac dysfunction by promoting myocardial fibrosis, disrupting capillary networks, and inducing pro-inflammatory M1 polarization of macrophages. In the kidney, MMP-9 directly drives renal fibrosis by mediating EMT and endothelial-mesenchymal transition.^65^ Integrin-linked kinase (ILK), a pivotal hub in ECM signaling, perceives ECM changes via integrins and activates multiple downstream signaling pathways to drive cardiac and renal injury. ILK deficiency promotes renal inflammation and fibrosis by activating the MLKL/RIPK3-mediated necroptosis pathway, while inhibiting this death pathway significantly improves renal outcomes.^66^ ILK-knockout mouse models further confirm that its absence also leads to a significant reduction in ureteric bud branching and defects in collecting duct formation.^67^ The network comprising ILK, integrin β1, and β-parvin functions as a cardiac stretch sensor.^68^ ILK deficiency or mutation disrupts signal transduction, triggering cardiomyocyte depolarization,^69^ cardiac hypertrophy, and impaired contractile function.^70^ Furthermore, macrophages secrete galectin-3, which synergistically activates fibroblasts with TGF-β, leading to massive synthesis and release of procollagen I into the ECM, ultimately triggering CRS fibrosis.^71^

## Characteristics of intercellular communication in specific CRS subtypes

Although the various subtypes of CRS share a common basis for intercellular communication (such as interactions mediated by soluble factors, EVs, and ECM), they differ in their etiology, disease course, and the organs primarily affected. Consequently, there are variations in the pathological signaling pathways and key molecular mechanisms between the heart and kidneys. These are described below according to each subtype.

### Type 1 cardiorenal syndrome

As a subtype characterized by acute kidney injury secondary to acute cardiac injury, type 1 CRS exhibits a distinctive “rapid response” pattern in intercellular communication. This response primarily relies on the synergistic effects of excessive hormone activation and acute inflammatory storms. Acute cardiac dysfunction (e.g., acute myocardial infarction, severe arrhythmias) causes a sharp decline in cardiac output, reduced renal perfusion pressure, and decreased renal blood flow and further activates RAAS. Once activated, angiotensin II—as the core signaling molecule—induces vasoconstriction, hypertrophy of cardiomyocytes and renal tubular cells, and fibrosis via paracrine effects.^72^ Aldosterone promotes secretion of galectin-3 by macrophages in cardiac and renal tissues, which in turn stimulates fibroblasts to secrete procollagen I and III, accelerating fibrosis in target organs.^6^^,^^73^ With regard to inflammation and immune regulation, studies have shown that activation of Toll-like receptors leads to the recruitment of monocytes/macrophages and neutrophils to the kidneys and promotes the massive release of pro-inflammatory cytokines such as tumor necrosis factor alpha (TNF-α), IL-1, and IL-6.^74^ These inflammatory mediators not only directly impair cardiomyocyte function and microvascular permeability,^75^ triggering pulmonary edema and reducing renal perfusion pressure, but also directly cause peritubular edema, decreased glomerular filtration rate, and tubular structural damage.^6^^,^^76^^,^^77^ Further studies reveal that in acute decompensated heart failure models, an IL-1β-centered inflammatory cytokine network serves as a key mechanism driving renal injury manifestations such as tubular damage, mesangial cell proliferation, and interstitial fibrosis.^78^

### Type 2 cardiorenal syndrome

Unlike the acute, rapid injury associated with type 1, type 2 CRS is characterized by chronic kidney injury secondary to chronic heart failure, with tissue fibrosis as its core pathological feature, and is driven by a combination of chronic inflammation and epigenetic regulation.^79^ Animal models demonstrate that renal fibrosis emerges early after myocardial infarction and progressively worsens with heart failure progression.^80^ Findings reveal persistent monocyte/macrophage infiltration throughout this process, and depletion of these cells significantly attenuates fibrosis,^81^ indicating that sustained immune inflammation drives fibrosis and constitutes the key mechanism of CRS injury.^80^ Research confirms that cardiac injury promotes the release of proinflammatory factors such as TNF-α into the bloodstream by activating the Wnt/β-catenin pathway.^82^ Circulating TNF-α suppresses the expression of the protective Klotho protein in the kidneys.^71^ Downregulation of Klotho releases inhibition on β-catenin signaling, leading to abnormal activation of β-catenin. Activated β-catenin ultimately drives irreversible renal fibrosis by upregulating key effector molecules of fibrosis, such as Snail1.^82^ Simultaneously, the TNF-like weak inducer of apoptosis (TWEAK) signaling pathway plays a synergistic role in promoting inflammation and fibrosis in both the heart and kidneys: in the heart, it induces fibroblasts to produce more collagen by activating the ras homolog family member A (RhoA) and nuclear factor κB (NF-κB) signaling pathways, thereby promoting cardiac fibrosis; in the kidneys, TWEAK acts on tubular cells, stimulating proliferation and upregulating proinflammatory factors (such as MCP-1 and IL-6) by synergistically activating the NF-κB pathway, extracellular-signal-regulated kinase (ERK) pathway, and phosphatidylinositol 3-kinase/protein kinase B (PI3K/Akt) pathway, thereby impacting renal function.^7^^,^^82^

Beyond the aforementioned pro-inflammatory fibrosis-related damage, epigenetic regulation—particularly the dysregulation of miRNAs—also plays a pivotal role in the CRS process. For example, in type 2 CRS mouse models, *miR-215-5p* is significantly downregulated.^83^ It inhibits epithelial-mesenchymal transition by suppressing the expression of zinc finger E-box binding homeobox 2, thereby preventing apoptosis and mitigating renal injury.^84^^,^^85^ Furthermore, studies indicate circular RNAs may influence cardiorenal cell-cell interactions by regulating hematopoietic cell lineages and cell adhesion molecule pathways, with *hsa_circ_0001763* offering novel molecular insights into type 2 CRS intercellular communication mechanisms.^86^

### Type 3 cardiorenal syndrome

In contrast to the “cardiac injury leading to renal damage” seen in type 1, type 3 CRS features a pathological process of acute cardiac injury secondary to acute kidney injury. Its pathogenesis centers on mitochondrial dysfunction induced by inflammatory cytokines.^87^ Additionally, renal injury signals are transmitted to the heart through multiple metabolic pathways.^88^ Sumida et al.^89^ established a mouse model of renal ischemia-reperfusion injury. They observed mitochondrial fragmentation in the heart 24 h post-surgery, activation of myocardial cell apoptosis at 72 h, and impaired left ventricular systolic function. Post-ischemia, cardiac tissue exhibited upregulation of dynamin-related protein 1 (Drp1) and TNF-α, and Drp1 inhibitors were found to mitigate mitochondrial fragmentation.^90^ At the mechanistic level, during AKI, inflammatory mediators like IL-6 act on the heart, upregulating growth factor receptor-bound protein 2 (Grb2) in cardiomyocytes. Grb2 impairs myocardial mitochondrial metabolism and ATP production by inhibiting the Akt signaling pathway,^91^ ultimately causing cardiac dysfunction.^92^

Additionally, the altered metabolic environment resulting from acute kidney injury directly impairs cardiac function. Uremic toxins serve as hallmarks of AKI. Research indicates that dimethylarginine, a small-molecule uremic toxin, correlates with endothelial dysfunction, vascular injury, and pro-inflammatory pathways. Moreover, infusion of asymmetric dimethylarginine into healthy individuals leads to reduced cardiac output and increased systemic vascular resistance.^93^ Under uremic conditions, exosomes secreted by macrophages infiltrating the heart carry *miR-155*. Upon uptake by cardiomyocytes, these exosomes target and suppress forkhead box O3a (FoxO3a) protein expression, activating the caspase-1/gasdermin D pathway to induce cardiomyocyte pyroptosis while simultaneously promoting myocardial hypertrophy and fibrosis.^94^ Furthermore, metabolic acidosis is a common complication of AKI. Acidosis directly impairs cardiac function by inducing systemic arteriolar vasodilation, arrhythmias,^95^ and reduced myocardial contractility.^96^ The underlying pathophysiology involves intracellular acidosis disrupting electrolyte currents associated with myocardial action potentials and altering intracellular calcium handling.^97^^,^^98^

### Type 4 cardiorenal syndrome

In type 4 CRS, intercellular communication is primarily driven by hyperphosphatemia and the accumulation of uremic toxins caused by chronic kidney disease, which drive cardiorenal cross-injury through pathways such as exacerbated anemia, fibrosis, and epigenetic mechanisms. Chronic kidney disease leads to phosphate accumulation in the blood. Excess phosphate enters cardiomyocytes via type III sodium-phosphate cotransporter 1/2 transporters, triggering histone H3 lysine 9 acetylation and subsequently upregulating the expression of the transcription factor interferon regulatory factor 1 (IRF1). The upregulated IRF1 protein inhibits the promoter of peroxisome-proliferator-activated receptor γ coactivator 1α (PGC1α), a core regulator of mitochondrial energy metabolism, resulting in a significant decrease in PGC1α expression. The loss of PGC1α downstream effects suppresses gene expression related to oxidative phosphorylation and fatty acid oxidation, triggering myocardial mitochondrial dysfunction, insufficient ATP production, and reactive oxygen species accumulation,^99^ ultimately driving ventricular hypertrophy and heart failure. On the other hand, hyperphosphatemia following chronic kidney disease jointly inhibits erythropoietin synthesis by suppressing vitamin D activation, elevating parathyroid hormone, and increasing fibroblast growth factor 23,^100^ while directly damaging cardiomyocytes. This creates a vicious cycle that exacerbates anemia^101^ and cardiovascular pathology.^102^

Furthermore, circulating miRNAs play a crucial role in type 4 CRS.^103^^,^^104^ During renal insufficiency, the uremic toxin indoxyl sulfate (IS) accumulates in the body and upregulates the expression of the pro-fibrotic *miR-21*, while reducing IS levels reverses myocardial fibrosis.^105^ In chronic kidney disease, *miR-155* induces cardiomyocyte pyroptosis by directly targeting FoxO3a, promoting the development of uremic cardiomyopathy.^94^ In chronic hemodialysis patients, lower blood *miR-133a* levels correlate with increased likelihood of left ventricular hypertrophy. Furthermore, *miR-133a* significantly reduces expression of activated nuclear factor of activated T cells cytoplasmic 4 (NFATc4) protein, negatively regulating cardiomyocyte hypertrophy.^85^^,^^106^

### Type 5 cardiorenal syndrome

Unlike the aforementioned four subtypes characterized by “single-organ injury leading to secondary damage in another organ,” type 5 CRS is defined by “simultaneous systemic injury to both the heart and kidneys.” Sepsis, as the core trigger of type 5 CRS,^107^ drives a vicious cycle of cardiac and renal injury through systemic inflammatory storms and endotoxin effects.^108^ In the heart, proinflammatory mediators and complement factors directly disrupt myocardial structural proteins and membrane-stabilizing proteins, leading to sepsis-associated cardiomyopathy; sepsis-associated acute kidney injury manifests as early proinflammatory factors and oxidative stress markers directly damaging renal tubules, while lipopolysaccharides exacerbate albuminuria and renal inflammation by interfering with HCO_3_^−^ transport and modifying podocyte proteins.^109^^,^^110^ Endotoxin, a major component of Gram-negative bacterial cell walls, plays a pivotal role in sepsis pathogenesis. Endotoxin directly acts on cardiomyocytes, disrupting calcium homeostasis and mitochondrial function while activating immune cells to release proinflammatory factors (e.g., TNF-α and IL-1β),^111^ inducing cardiomyocyte apoptosis and ventricular dilatation. It also triggers renal shunting and intrarenal vascular resistance imbalance by activating Toll-like receptor 4 and can even directly induce tubular cell apoptosis.^112^

In addition to sepsis, metabolic diseases such as diabetes, obesity, and hypertension simultaneously damage both the heart and kidneys through a series of molecular pathways.^113^ For example, the pathological process of HF in diabetic nephropathy is closely associated with the accumulation of advanced glycation end products (AGEs). AGEs form abnormal cross-links in myocardial interstitium, myofibrils, and glomerular filtration structures, promoting collagen deposition in cardiac and renal interstitium. This leads to tissue stiffening and functional impairment,^114^ indicating that AGE-mediated collagen cross-linking plays a pivotal role in the development of diabetic cardiorenal comorbidity.^115^ Similarly, in systemic lupus erythematosus (SLE), CD177^+^ neutrophils enhance NET formation via ROS-dependent pathways, induce endothelial apoptosis, and drive cardiovascular damage.^116^ Knocking out the CD177 gene alleviates renal injury^117^ and vascular dysfunction^116^ in lupus mice, confirming the central role of this immune cell subset in concurrent cardiac and renal injury.

## Therapeutic strategies and challenges targeting intercellular communication

### New mechanistic interpretation of existing drugs

Current drugs for treating CRS can be reinterpreted from the novel perspective of “dysregulated intercellular communication,” providing more precise theoretical support for rational clinical drug use. Specifically, different drugs mitigate the state of heart-kidney mutual impairment by synergistically alleviating communication abnormalities across multiple levels—including neurohumoral, secretory factors, and signaling molecules—through targeted interventions (Table 1).

**Table 1:** Strategies for targeting cardiorenal communication in the intervention of CRS

Intervention category	Specific strategies/drugs	Core mechanism of action	Cardiorenal protective effects
Existing drugs—RAAS inhibitors	angiotensin-converting enzyme inhibitors (ACEIs; e.g., captopril)	inhibit the production of Ang Ⅱ and block the excessive activation of the RAAS	reduce cardiac afterload and improve left ventricular diastolic function; relieve renal vasoconstriction, increase renal blood flow, and reduce proteinuria
Existing drugs—RAAS inhibitors	Ang Ⅱ receptor blockers (ARBs; e.g., losartan)	competitively inhibit the binding of Ang Ⅱ to AT1 receptors and block the excessive activation of the RAAS	restore renal vascular filtration function; interrupt the malignant communication cycle between renal blood vessels and the sympathetic nervous system
Existing drugs—RAAS inhibitors	angiotensin receptor neprilysin inhibitors (ARNIs; e.g., sacubitril/valsartan)	inhibit TRPC6–NFATc–Rcan1, TGF-β1/Smad3, and Wnt/β-catenin pathways; regulate mitofusin 2 (Mfn2) and cellular stress signaling pathways	reverse the flattening of renal podocyte foot processes; reduce myocardial fibrosis; protect the structural and functional integrity of cardiomyocytes and renal parenchyma
Existing drugs—RAAS inhibitors	novel ARNI (e.g., S086)	antagonize ARB and inhibit neprilysin, enhance natriuretic peptide system activity	regulate the expression of fibrosis-related factors (MMP-2, α-SMA, and TGF-β1) and reduce cardiorenal remodeling
Existing drugs—mineralocorticoid receptor antagonists (MRA)	non-steroidal MRA (e.g., finerenone)	inhibit the transcription of pro-fibrotic factors (TGF-β1) and block the inflammation-fibrosis communication driven by excessive MR activation	slow the progression of chronic kidney disease and reduce the risk of cardiovascular events; block the inflammation-fibrosis process of cardiorenal tissues
Existing drugs—Metabolic and energy regulators	SGLT2 inhibitors	alleviate renal tubular metabolic load; promote the conversion of cardiomyocyte metabolic substrates to ketone bodies; activate Sirtuin-1, selectively up-regulate HIF-2α, and inhibit HIF-1α	alleviate intrarenal metabolic stress, improve myocardial energy utilization efficiency, reduce renal tissue fibrosis and inflammatory response, alleviate hypoxic injury of cardiomyocytes
Existing drugs—second messenger system modulators	soluble guanylate cyclase (sGC) stimulants (e.g., riociguat)	enhance the activation of active sGC by NO, directly activate sGC inactivated by oxidative stress, increase cGMP levels	reduce cardiac preload and improve renal hemodynamics; reduce myocardial and renal fibrosis
Existing strategies—endogenous hormone targeted regulation	apela hormone	inhibit the expression of pro-inflammatory factors (MCP-1 and TNF-α) and adhesion molecules (ICAM-1 and VCAM-1), and reduce the adhesion between THP-1 monocytes and glomerular endothelial cells	alleviate renal inflammatory response, improve cardiorenal function, and provide a target for anti-inflammatory treatment of CRS
Existing strategies—cell-level targeted intervention	mesenchymal stem cell (MSC) transplantation	promote mitophagy and upregulate the SIRT1-Parkin axis (renal tissue)	promote mitophagy in renal tubular epithelial cells to alleviate AKI; alleviate high glucose-induced vascular endothelial cell death
emerging strategies—extracellular vesicles (EVs)	mesenchymal-stem-cell-derived exosomes (MSC-EV)	deliver functional proteins and miRNAs, activate the PI3K/Akt pathway (heart), regulate transcriptional processes and immune regulation (kidney)	reduce cardiac oxidative stress and improve cardiac function; promote renal tissue regeneration
Emerging strategies—engineered extracellular vesicles	engineered EVs with surface ligand modification/internal nucleic acid drug loading	enhance the targeted enrichment ability in cardiorenal injury areas and reduce off-target effects	enhance the repair effect on cardiorenal injury tissues and improve targeted treatment efficiency
Emerging strategies—pretreated MSC exosomes	MSC exosomes pretreated with hypoxic culture/cytokines/biomolecular stimulation/gene overexpression	enhance the angiogenesis, anti-inflammatory and immune regulation abilities of exosomes; achieve precise targeted modification of the heart and kidney	improve the repair and protection efficiency of exosomes on the heart and kidney organs
Emerging strategies—miRNA-targeted regulation	antisense oligonucleotides (e.g., RG-012)	directly silence pathogenic miRNAs (e.g., pro-fibrotic miR-21) after chemical modification	inhibit the process of cardiorenal fibrosis and alleviate chronic cardiorenal tissue injury
Emerging Strategies—miRNA-targeted regulation	nanocarrier-delivered miRNA/siRNA	nanoparticles deliver siRNAs targeting CCR2 and CSF-1; exosomes and other carriers deliver miR-5100 mimics	reduce myocardial inflammation and improve cardiac function; reduce renal tubular injury and inhibit intrarenal inflammation and cell apoptosis
Emerging strategies—nanodrugs	polylactic/glycolic acid nanoparticles as carriers for cyclosporine A	inhibit the opening of mitochondrial permeability transition pores	protect the heart
Emerging strategies—nanodrugs	selenium-bovine serum albumin nanomaterials (Se@BSA NPs)	up-regulate GPx-1, inhibit NLRP3 inflammasome activation	alleviate the inflammatory response of renal tubular epithelial cells and effectively relieve renal injury
Emerging strategies—omics technology assistance	single-cell RNA sequencing (scRNA-seq)	analyze cell heterogeneity and molecular networks in CRS and locate key pathogenic cell subsets, core genes, and signaling pathways	provide a theoretical basis for the development of precise targeted therapies at the cellular/molecular level and promote the research on precise intervention of CRS
Emerging strategies—neural-pathway-targeted regulation	paraventricular nucleus (PVN)-related neural regulation	the small-cell neurons in the paraventricular nucleus of the hypothalamus specifically regulate the heart and kidneys	precisely regulate the cardiorenal cross-organ neural communication and provide a new path for the precise intervention of CRS

With regard to CRS, RAAS inhibitors are currently the most widely used medications; they primarily exert their effects through neurohumoral regulation.^118^ Among these, angiotensin-converting enzyme inhibitors (ACEIs) and angiotensin II receptor blockers (ARBs) can effectively suppress the acute activation of the RAAS triggered by chronic heart failure, thereby reducing subsequent adverse reactions such as renovascular constriction. For example, captopril, by inhibiting Ang II production, not only reduces afterload on the heart and improves left ventricular diastolic function but also alleviates renal vasoconstriction, increases renal blood flow, and reduces proteinuria. Similarly, losartan competitively inhibits Ang II binding to AT1 receptors. In congestive heart failure models, it restores renal vascular filtration function and suppresses abnormal norepinephrine release from sympathetic nerve endings, thereby interrupting the vicious communication loop between the renal vasculature and sympathetic nervous system.^119^

Sacubitril/valsartan, a representative angiotensin receptor-neprilysin inhibitor (ARNI), effectively reverses the flattening of podocyte foot processes in the kidney by inhibiting the transient receptor potential canonical 6-nuclear factor of activated T cells cytoplasmic-regulator of calcineurin 1 (TRPC6–NFATc–Rcan1) pathway^120^ and prevents the loss of its key proteins nephrin and podocin^121^; simultaneously, it mitigates myocardial fibrosis by inhibiting TGF-β1/Smad3 and Wnt/β-catenin signaling pathways.^122^ In vivo studies indicate that sacubitril/valsartan primarily protects myocardial and renal parenchymal structural and functional integrity by regulating mitofusin 2 (Mfn2) and cellular stress signaling pathways while reducing oxidative stress generation.^123^ In recent years, the novel ARNI drug S086 has demonstrated enhanced efficacy by simultaneously antagonizing Ang II receptors and inhibiting neuropeptidase, thereby amplifying natriuretic peptide system activity. This mechanism enables more potent regulation of neuroendocrine communication networks, achieving sustained blood pressure reduction while modulating the expression of fibrosis-related factors such as MMP-2, α-smooth muscle actin (α-SMA), and TGF-β1, and alleviating cardiac and renal remodeling.^124^ According to current research, RAAS inhibitors are primarily used as first-line therapy for type 2 and type 4 CRS and may also be used as adjunctive therapy once type 1 and type 3 CRS have stabilized.

Mineralocorticoid receptor antagonists (MRAs) have also made new advances within this framework, demonstrating not only the ability to reduce proteinuria but also to slow CKD progression and mitigate cardiovascular events.^125^ The non-steroidal MRA finerenone exhibits significant cardiorenal protective effects^126^; it sustainably inhibits the transcription of pro-fibrotic factors (such as TGF-β1) even in the absence of aldosterone, thereby more safely and effectively blocking the inflammation-fibrosis communication driven by MR overactivation.^127^ It is applicable to type 2 and type 4 CRS, where chronic fibrosis is central.^128^ Furthermore, large-scale clinical trials confirm that finerenone significantly reduces cardiovascular and renal composite endpoint risks in patients with type 2 diabetes mellitus and CKD,^129^^,^^130^ delaying disease progression. Therefore, its use may also be considered for type 5 CRS.

In terms of metabolic and energy signaling regulation, sodium-glucose cotransporter 2 (SGLT2) inhibitors demonstrate particularly multifaceted therapeutic potential.^131^ By inhibiting SGLT2 in the proximal tubules, these agents not only reduce glomerular pressure but also alleviate tubular metabolic burden and suppress NLRP3 inflammasome activation, thereby alleviating renal metabolic stress.^132^ SGLT2 inhibitors also promote a shift in myocardial cell metabolic substrates from glucose to ketone bodies, improving energy utilization efficiency^133^; they exert cardiac effects by inhibiting myocardial Na+/H+ exchanger (NHE) activity, reducing cytoplasmic Na^+^ concentration, and inducing vasodilation.^134^ Another study indicates that SGLT2 inhibitors activate *Sirtuin-1* and selectively upregulate hypoxia-inducible factor 2-α (HIF-2α) while suppressing hypoxia-inducible factor 1-α (HIF-1α), thereby reducing renal tissue fibrosis,^135^ inflammatory responses, and myocardial cell hypoxic injury,^136^ achieving dual protection for the heart and kidneys.^137^ SGLT2 inhibitors demonstrate multifaceted efficacy beyond glucose control, primarily through improving cardiorenal metabolic communication and energy homeostasis. They provide clear cardiorenal benefits in type 2, type 4, and type 5 CRS with diabetes.^138^

Additionally, drugs that directly target intracellular second messenger systems offer new pathways for regulating cardiorenal communication. Soluble guanylate cyclase (sGC) stimulants enhance NO’s activation of active sGC and can directly reactivate sGC inactivated by oxidative stress, thereby elevating cyclic guanosine monophosphate (cGMP) levels.^139^ This subsequently activates protein kinase G (PKG), reducing cardiac preload and improving renal hemodynamics without risks such as hyperkalemia or stroke.^140^ The functional integrity of the cGMP-PKG pathway is critical for maintaining cardiac and renal homeostasis. Deficiencies in this pathway reduce myocardial myosin phosphoprotein phosphorylation, leading to increased myocardial stiffness.^140^ In the kidneys, it triggers interstitial fibrosis and tubular cell apoptosis.^141^ sGC stimulators exert their protective effects by restoring this pathway. Animal studies confirm that in both low-renin and high-renin rat models of hypertension, the sGC stimulator riociguat significantly reduces myocardial and renal fibrosis while improving cardiorenal injury.^142^ This makes it particularly suitable for treating type 2 and type 4 CRS, which are characterized by chronic fibrosis and vascular dysfunction.

In addition to the aforementioned classic signaling-pathway-targeted drugs that have been widely applied in clinical settings, recent years have also identified several endogenous candidate hormones and cellular-level intervention approaches with potential cardiorenal protective effects. For instance, the Apela hormone has been shown to alleviate renal inflammation and improve cardiorenal function. When Ang II stimulates glomerular endothelial cells to induce inflammation, Apela significantly suppresses multiple proinflammatory factors (e.g., MCP-1 and TNF-α) and adhesion molecules (e.g., intercellular adhesion molecule 1 [ICAM-1] and vascular cell adhesion molecule 1 [VCAM-1]), and reduces the adhesion of Tohoku Hospital Pediatrics-1 cell line monocytes to glomerular endothelial cells.^143^ This suggests that Apela offers a potential therapeutic target for CRS by counteracting inflammatory responses. Meanwhile, studies have shown that MSC transplantation can restore cellular function by promoting mitochondrial autophagy.^144^ In the kidney, MSCs upregulate the SIRT1-Parkin axis, promoting mitochondrial autophagy in renal tubular epithelial cells and alleviating AKI; in cardiovascular disease (CVD), MSCs reduce cell death by inhibiting the expression of Parkin and PTEN-induced putative kinase 1 in human umbilical vein endothelial cells exposed to high glucose levels.^145^ These studies suggest that MSCs may serve as a potential therapeutic strategy for CRS.^146^

### Emerging targeted strategies

Based on in-depth exploration and analysis of intercellular communication mechanisms in CRS, a series of emerging targeted strategies are transitioning from basic research to clinical translation. These new strategies primarily cover the following areas: extracellular vesicle therapy, miRNA-targeted delivery systems, nanomedicines, and single-cell RNA sequencing (scRNA-seq). Collectively, they maintain cardiac and renal homeostasis at multiple levels (Table 1).

First, extracellular vesicles, particularly exosomes, serve as endogenous nanoscale communication carriers.^147^^,^^148^ For instance, mesenchymal-stem-cell-derived exosomes can alleviate oxidative stress in the heart and activate the PI3K/Akt pathway to improve cardiac function by delivering functional proteins and miRNAs. In the kidney, they regulate transcriptional processes and immunomodulation to promote tissue regeneration.^149^ To further enhance the targeting specificity and therapeutic efficacy of EVs, functional enhancement can be achieved through engineered modifications. These primarily involve surface ligand modification and internal loading of specific nucleic acid therapeutics (such as small interfering RNA [siRNA] or miRNA mimics), thereby improving their targeted accumulation and therapeutic efficiency in areas of cardiac and renal injury. This enables precise regulation of the microenvironment while reducing off-target effects.^150^^,^^151^ Additionally, since the physiological state of MSCs directly influences exosome characteristics, their pretreatment is crucial for enhancing exosome yield and function. Key strategies include enhancing their angiogenic and reparative capabilities through hypoxic culture; strengthening anti-inflammatory and immunomodulatory functions via cytokine (e.g., IFN-γ and TNF-α) or biomolecular (e.g., lipopolysaccharide and melatonin) stimulation; and achieving precise engineered modifications through gene overexpression (e.g., GATA binding protein 4, specific miRNAs) to specifically enhance therapeutic efficacy for organs such as the heart and kidneys.^152^

Second, miRNA-targeting strategies hold great promise due to their central role in gene expression, yet their functions exhibit spatiotemporal specificity across disease stages and organs. For instance, *miR-21* promotes cardiorenal fibrosis in chronic disease phases but may exert protective effects during acute phases.^153^ This complexity demands precise and controllable intervention strategies. Current primary strategies include the following: first, directly silencing pathogenic miRNAs using chemically modified molecular drugs such as antisense oligonucleotides^104^ (e.g., RG-012 targeting pro-fibrotic *miR-21* has entered clinical trials^153^); second, employing novel nanocarrier systems (e.g., liposomes, polymers, or engineered exosomes) to deliver targeted miRNA (e.g., *miR-126*)^153^^,^^154^ or siRNA therapeutics, precisely silencing pro-inflammatory genes or supplementing beneficial molecules to synchronously coordinate cardiac and renal cellular responses. For instance, in acute cardiac injury, nanoparticle delivery of siRNA targeting chemokine C-C motif receptor 2 (CCR2) and colony-stimulating factor 1 (CSF-1) reduces myocardial inflammation and improves cardiac function; in acute kidney injury, exosome-based carriers delivering *miR-5100* mimics reduce tubular damage while suppressing renal inflammation and apoptosis,^155^ offering novel directions for targeted CRS intervention.

In addition, nanomedicines, with their nanoscale carrier structures, can significantly enhance drug targeting, stability, and delivery efficiency. Polymer nanoparticles combine good biocompatibility with controlled-release properties.^156^ In one study, polylactic/glycolic acid nanoparticles were used as a carrier for cyclosporine A, exerting a cardioprotective effect by inhibiting the opening of mitochondrial permeabilization pores.^157^ Additionally, selenium-conjugated bovine serum albumin nanomaterials (Se@BSA NPs) alleviate inflammatory responses in renal tubular epithelial cells by upregulating glutathione peroxidase 1 (GPx-1) and inhibiting NLRP3 inflammasome activation.^158^ Although the application of nanomaterials in CRS has not yet become widespread, the aforementioned studies provide potential new strategies for mitigating cardiac and renal injury in CRS.

It is worth noting that high-resolution omics technologies, such as scRNA-seq, are providing in-depth insights into cellular heterogeneity and molecular networks in CRS, offering critical evidence for the discovery and optimization of targeted therapeutic strategies. For example, scRNA-seq combined with immune infiltration analysis has confirmed that macrophages are key cells in CRS-induced renal fibrosis, and their key gene, HIF-1α, mediates the polarization of macrophages toward the M1 phenotype, activates the PKM2/mTORC1/YME1L signaling axis within peritubular cells, drives epithelial-mesenchymal transition (EMT) and mitochondrial metabolic reprogramming, and ultimately leads to renal fibrosis in CRS.^159^ Furthermore, Kevin G. Burfeind et al. revealed through scRNA-seq that the S1 and S2 segments of the proximal tubules and cells of the distal tubules in the kidneys of mice with acute CRS exhibit the most significant damage and are enriched with immune-related pathways, providing critical evidence at the single-cell level for mechanistic studies and target development in CRS.^160^

Beyond the aforementioned molecular and carrier strategies, neural pathways provide a rapid conduit for cardiorenal communication. Recent evidence indicates bidirectional neural connections between the kidneys and heart, particularly via small-cell neurons in the paraventricular nucleus (PVN) of the hypothalamus. These neurons not only specifically regulate cardiac and renal functions but also project to sympathetic drive centers such as the rostral ventrolateral medulla (RVLM) and the intermediolateral column of the spinal cord, thereby modulating overall sympathetic tone and cardiovascular function. Neuroanatomical studies further confirm close spatial association between PVN axon terminals and RVLM neurons, with some terminals directly innervating preganglionic sympathetic neurons of the heart and kidneys,^161^ providing a structural basis for this cross-organ neural regulatory mechanism. This discovery holds promise for pioneering new directions in precision interventions for CRS.

### Challenges faced

Although targeting intercellular communication offers a new approach for CRS intervention, its clinical translation still faces numerous challenges. At the mechanistic level, the cardiorenal communication network exhibits high specificity, with the same molecule potentially exerting opposite functions at different stages. For example, *miR-21* inhibits apoptosis during the acute phase but promotes fibrosis during the chronic phase.^153^ Cardiorenal communication involves multiple mechanisms, including soluble factors, EVs, and the ECM; however, systematic elucidation of the regulatory nodes and upstream-downstream relationships remains lacking. Furthermore, while the communication characteristics vary across different subtypes of CRS, core molecules overlap, making it difficult to precisely distinguish specific regulatory patterns. Technologically, achieving efficient and specific delivery of therapeutic molecules remains a major bottleneck. Although exosomes and nanocarriers exhibit good biocompatibility,^162^ their in vivo targeting efficiency, loading capacity, and controlled release are far from meeting clinical demands.^163^ Natural exosomes tend to accumulate in the liver and exhibit weak chemotaxis toward the heart and kidneys^164^^,^^165^; although their surfaces can be modified via genetic engineering to enhance directed delivery,^166^ this may disrupt the vesicle membrane structure, reduce drug-loading efficiency, and pose immunological risks.^167^ While chemical modification of oligonucleotide drugs improves stability, it may lead to off-target effects and organ toxicity.^168^ At the clinical translation level, the heterogeneity and complexity of CRS pose deeper challenges for intervention. The patient population often consists of elderly individuals with multiple chronic conditions and complex medication histories, and the disease itself is classified into five subtypes, each potentially driven by distinct signaling mechanisms. However, existing preclinical models struggle to adequately simulate this dynamic progression and the in vivo environment, resulting in limited predictive value. Furthermore, large-scale production, standardized quality control, and stable storage protocols for exosomes and nanomedicines have yet to be established, and their long-term safety and individual variability require more rigorous evaluation systems.^169^

## Conclusions

Intercellular communication plays a central role in the onset and progression of CRS, and its finely tuned regulatory network offers a new perspective for overcoming therapeutic bottlenecks. Future research directions may focus on the following areas: (1) systematically elucidating the functions of non-coding RNAs in CRS and their regulatory networks, clarifying their spatiotemporal specificity as intercellular signaling mediators^170^; (2) focusing on the specific molecular mechanisms of intercellular communication in different CRS subtypes to identify subtype-specific biomarkers and regulatory nodes; (3) deepening research into the mechanisms of EV-mediated cross-organ communication and advance targeted modification and large-scale production technologies for engineered therapeutic EVs; (4) achieving cell-specific targeted gene therapy using diverse delivery vehicles; (5) integrating single-cell sequencing^171^ with spatial transcriptomics^160^ to map the dynamic landscape of heart-kidney interactions; and (6) combining artificial intelligence with integrated analysis of multi-omics data to enable personalized assessment. Ultimately, we aim to facilitate the translation of basic research into clinical practice by conducting prospective clinical trials targeting specific signaling molecules or pathways, developing precision treatment strategies tailored to different CRS subtypes, and laying the foundation for the transition from decoding “cellular communication” to implementing “targeted interventions.”

## Limitations of the review

This review focuses on the intercellular communication mechanisms and therapeutic advances in cardio-renal syndrome. Although it systematically summarizes relevant research, the following limitations remain. First, the conclusions of this review are based on published literature, which may be subject to selection bias and may not comprehensively cover all relevant research findings worldwide. Second, existing studies exhibit significant heterogeneity in experimental models, sample sizes, and detection methods, making it difficult to conduct a unified quantitative analysis and thereby affecting the consistency of the conclusions. Finally, emerging therapeutic strategies remain in the basic research phase and lack support from large-scale clinical data; their feasibility and safety for clinical application require further validation.

## Acknowledgments

This work was supported by the 10.13039/501100001809National Natural Science Foundation of China (81603559), China Heart House-Chinese Cardiovascular Association-2024 TCM fund（2024-CCA-TCM-005, and Hebei Province Traditional Chinese Medicine Scientific Research Project Program (T2025058). We sincerely acknowledge the financial support from the aforementioned funding sources. Additionally, we thank the affiliated institutions of all authors for providing necessary research resources and technical support. Finally, we appreciate the valuable suggestions from the reviewers and editors during the submission process, which have significantly enhanced the quality and rigor of this manuscript.

## Author contributions

S.L. was responsible for conceptualization and research design, M.W. wrote the initial draft and handled major revisions, Y.C. collected data and participated in major revisions, L.W. was responsible for data collection and assisted with revisions, A.L. assisted with manuscript revisions and provided key guidance, and H.Z. collected data. J.C., Y.K., Z.Y., and G.S. performed literature collation. L.F. and J.Z. offered insights, supplements to the content, and guidance on figure preparation. X.L. was responsible for project administration, resource support, manuscript review, and editing. X.W. and Y.L. supervised the project and coordinated the revisions. All authors discussed the content collectively, contributed to the manuscript revisions, and approved the final version.

## Declaration of interests

The authors declare no competing interests.
